# Supplementary material for: Endothelial Lipase Modulates Paraoxonase 1 Content and Arylesterase Activity of HDL
Source: Int J Mol Sci. 2021 Jan 13;22(2):719. doi: 10.3390/ijms22020719 (PMC7828365; doi:10.3390/ijms22020719)
Supplement: Supplementary file 1 [file ijms-22-00719-s001.zip › Original WB.pdf]

A & D

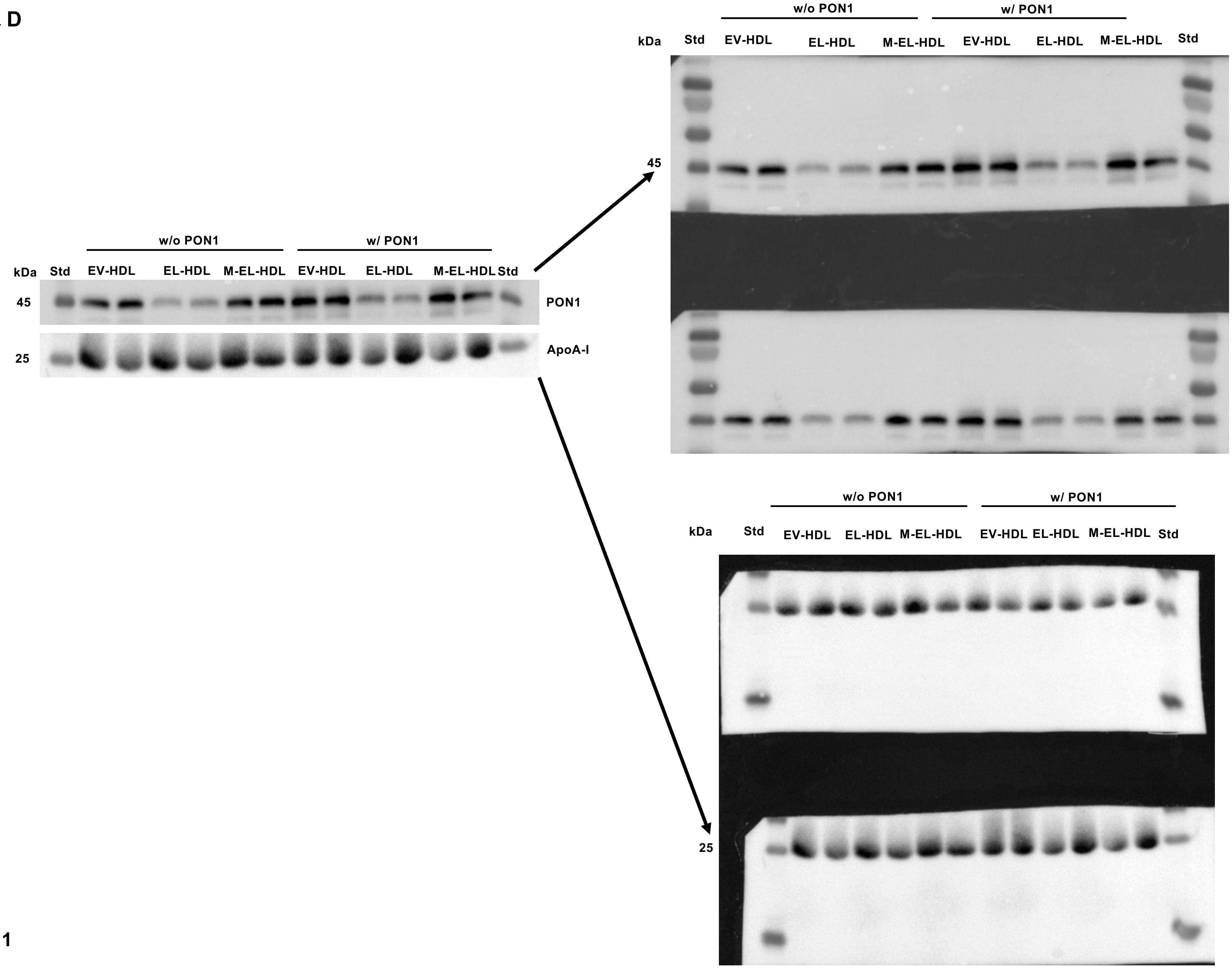
w/o PON1
w/ PON1
kDa
Std
EV-HDL
EL-HDL
M-EL-HDL
EV-HDL
EL-HDL
M-EL-HDL
Std
45
25
PON1
ApoA-I

Fig. 1

C

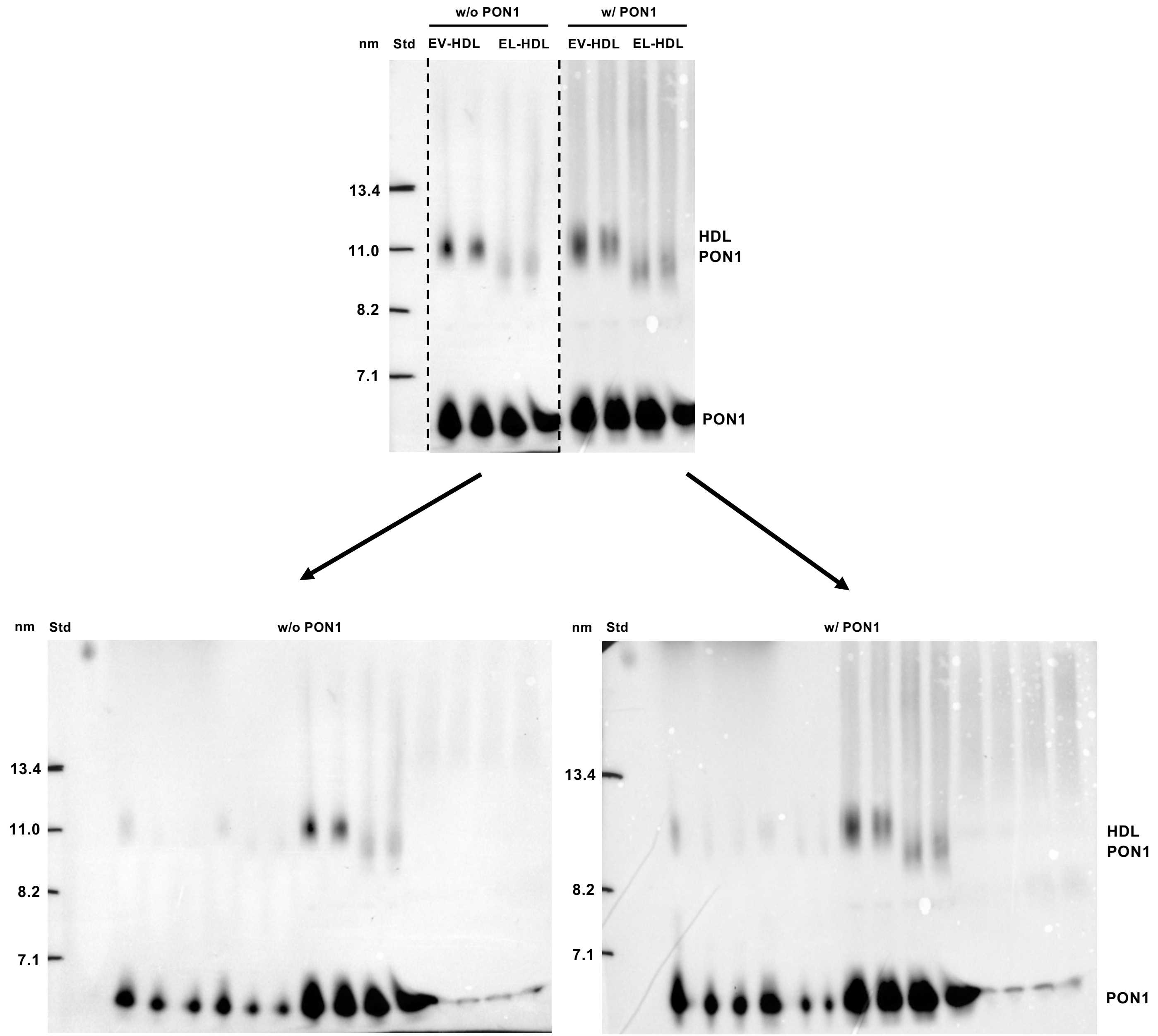

Lane	Sample	
1	Std	
2	EV-HDL w/o BSA	Isolated HDL
3/4	EL-HDL w/o BSA	Isolated HDL
5	EV-HDL w/ BSA	Isolated HDL
6/7	EL-HDL w/ BSA	Isolated HDL
8/9	EV-HDL w/o BSA	Modified medium
10/11	EL-HDL w/o BSA	Modified medium
12/13	EV-HDL w/ BSA	Modified medium
14/15	EL-HDL w/ BSA	Modified medium

Fig. 1

A

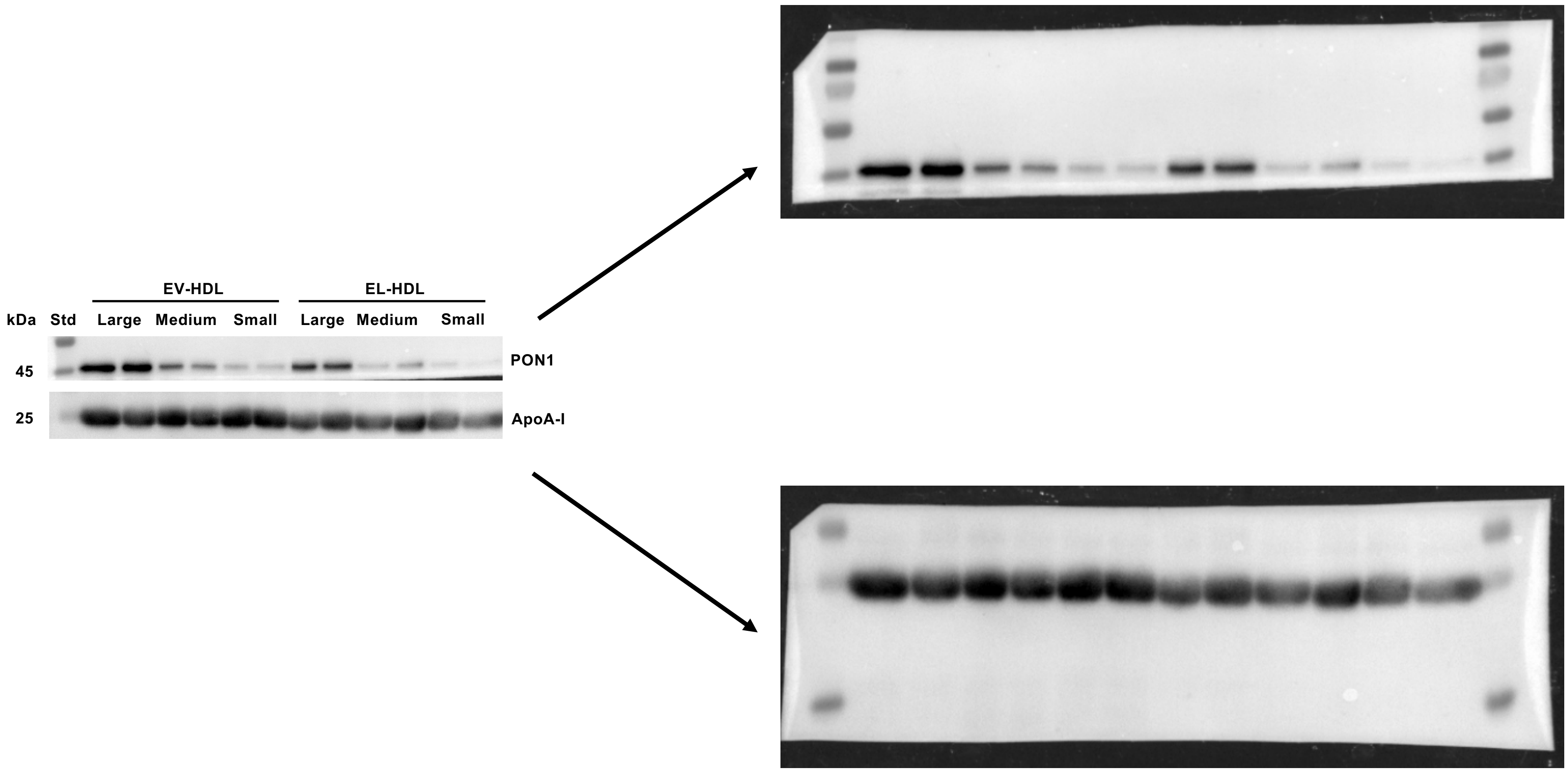
EV-HDL
EL-HDL
kDa
Std
Large
Medium
Small
Large
Medium
Small
45
25
PON1
ApoA-I

Fig. 2

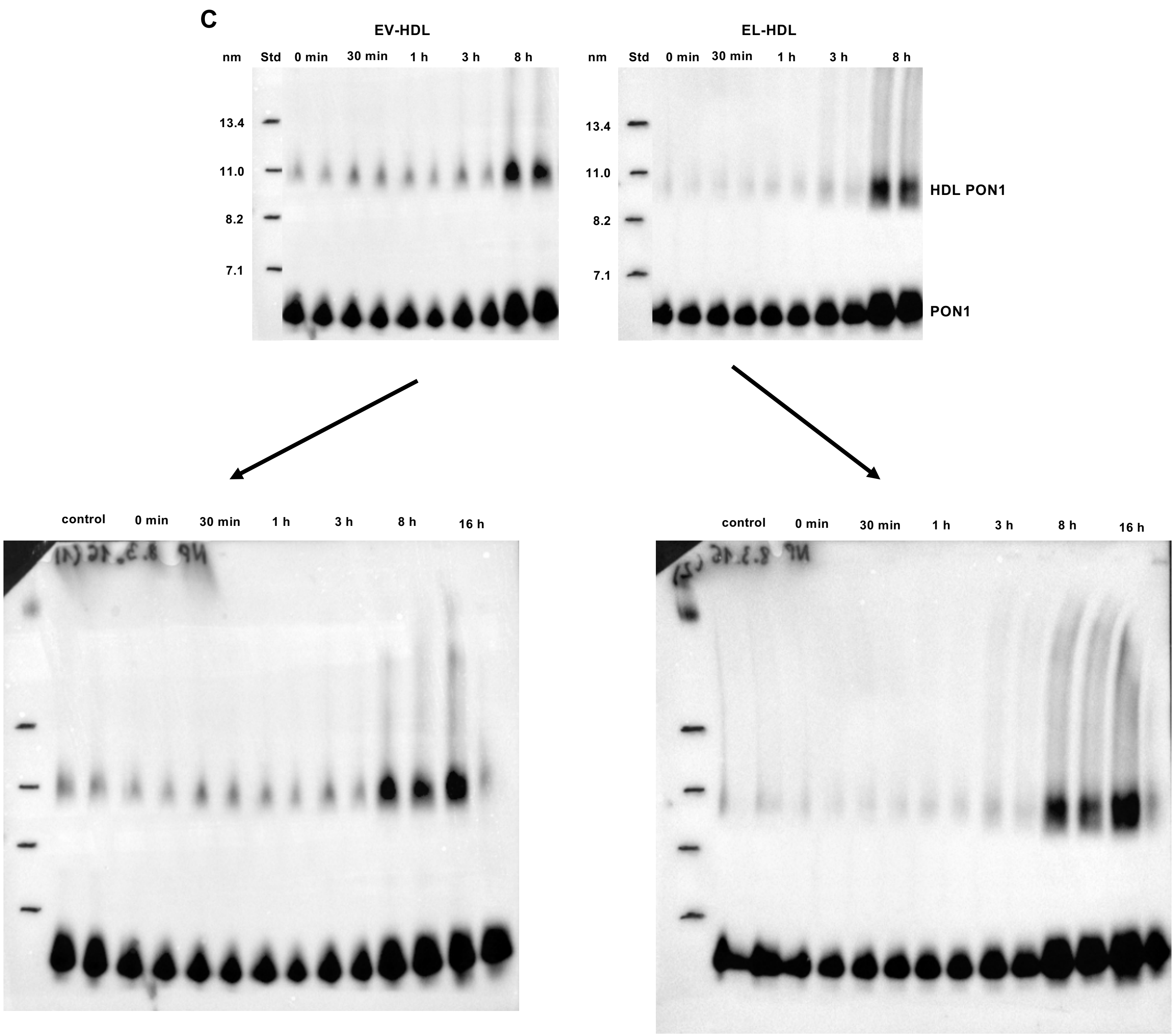
C
EV-HDL
EL-HDL
nm
Std
0 min
30 min
1 h
3 h
8 h
13.4
11.0
8.2
7.1
HDL PON1
PON1
control
16 h

Fig. 2

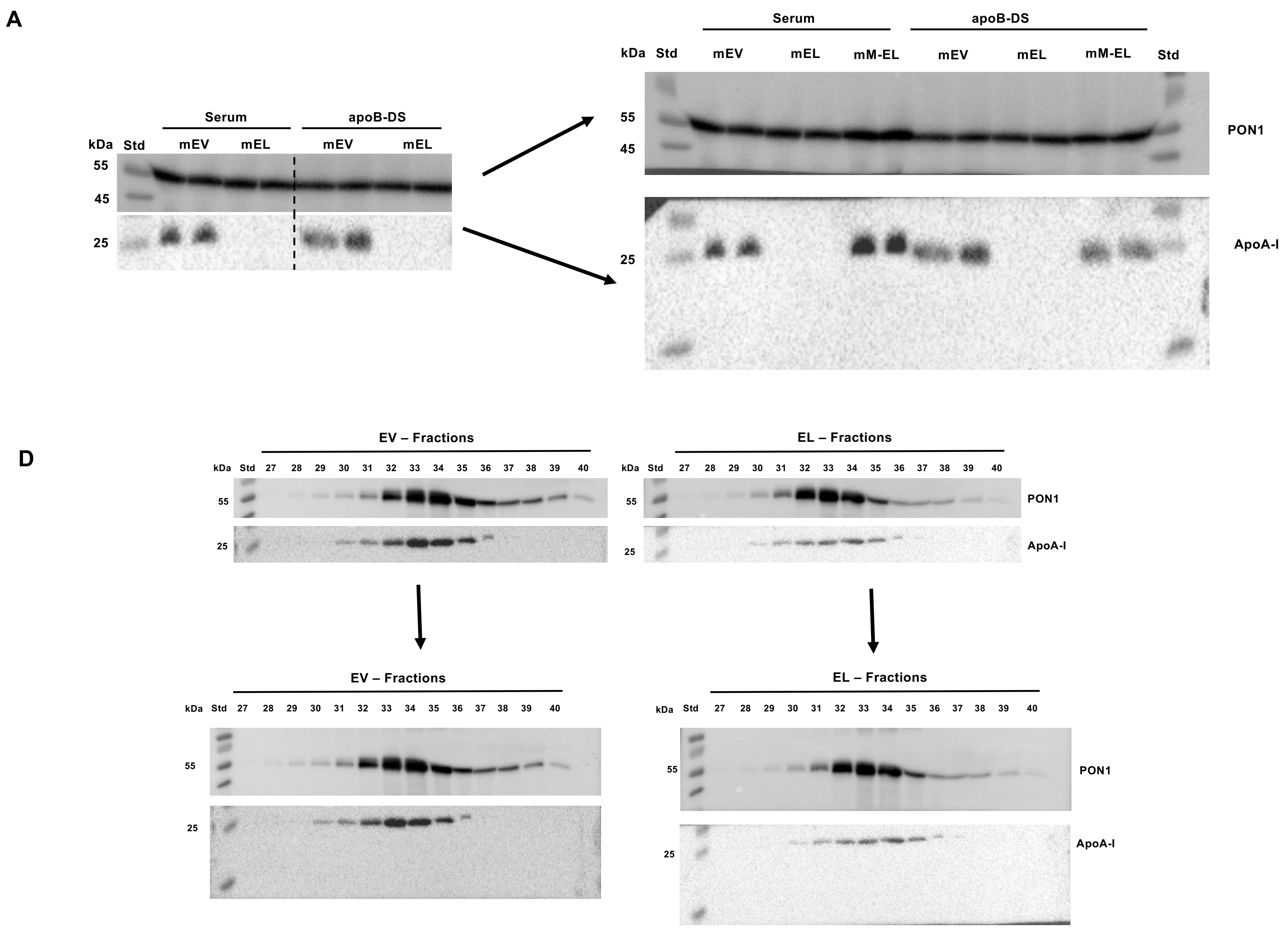
A
Serum
apoB-DS
kDa
Std
mEV
mEL
55
45
25
Serum
apoB-DS
kDa Std mEV mEL mM-EL mEV mEL mM-EL Std
55
45
PON1
25
ApoA-I
D
EV – Fractions
kDa Std 27 28 29 30 31 32 33 34 35 36 37 38 39 40
55
25
EL – Fractions
kDa Std 27 28 29 30 31 32 33 34 35 36 37 38 39 40
55
25
PON1
ApoA-I
EV – Fractions
kDa Std 27 28 29 30 31 32 33 34 35 36 37 38 39 40
55
25
EL – Fractions
kDa Std 27 28 29 30 31 32 33 34 35 36 37 38 39 40
55
25
PON1
ApoA-I

Fig. 3

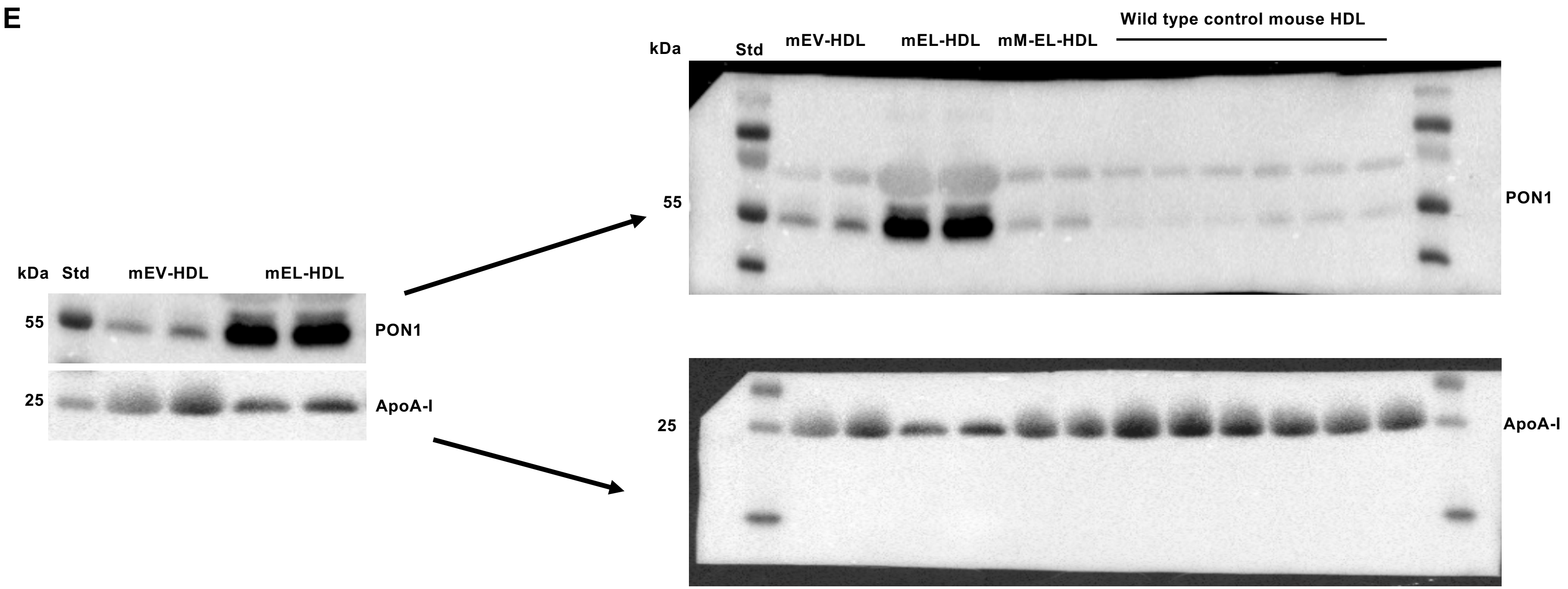
E
kDa
Std
mEV-HDL
mEL-HDL
55
PON1
25
ApoA-I
kDa
Std
mEV-HDL
mEL-HDL
mM-EL-HDL
Wild type control mouse HDL
55
PON1
25
ApoA-I

Fig. 3

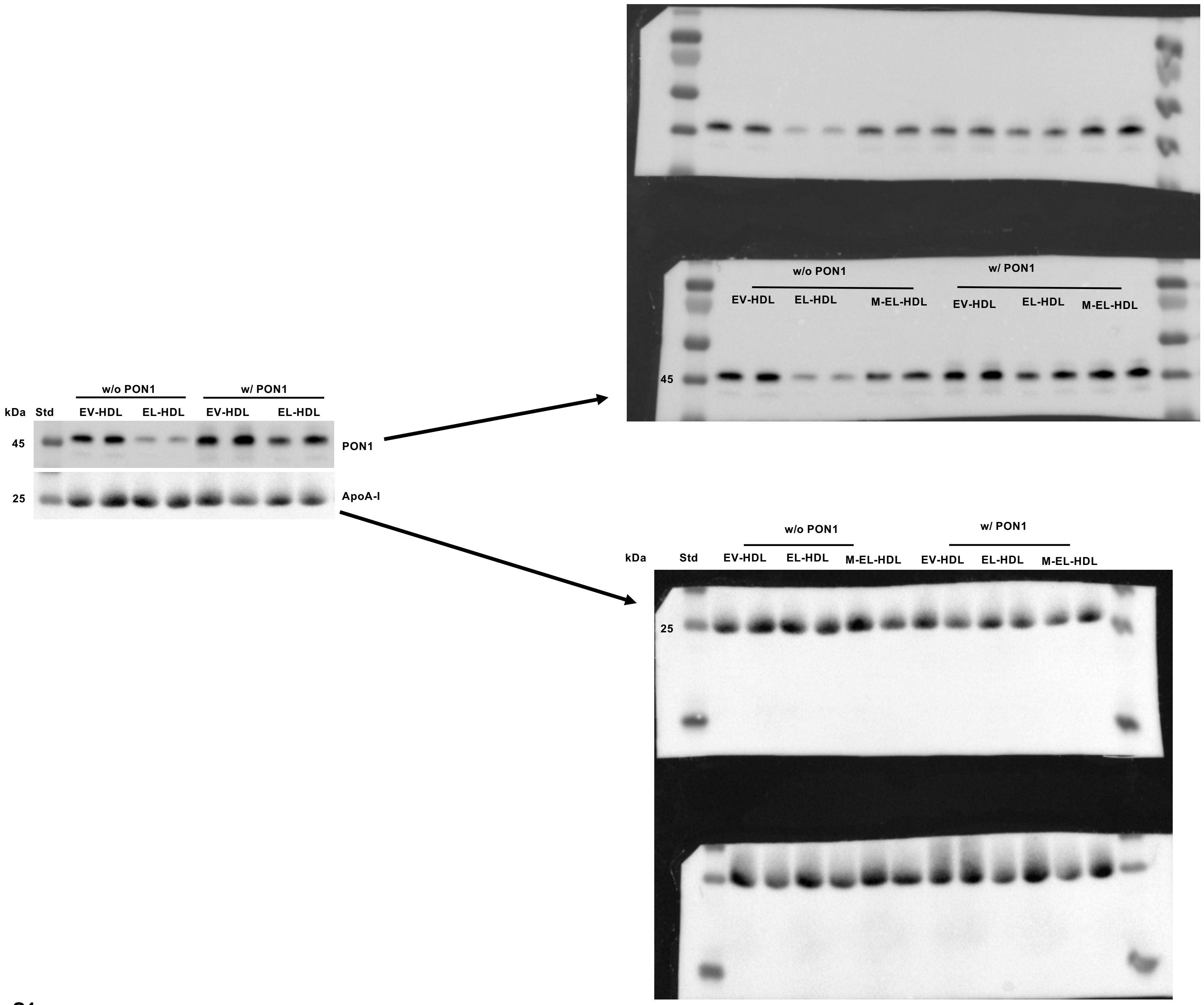

Figure S1

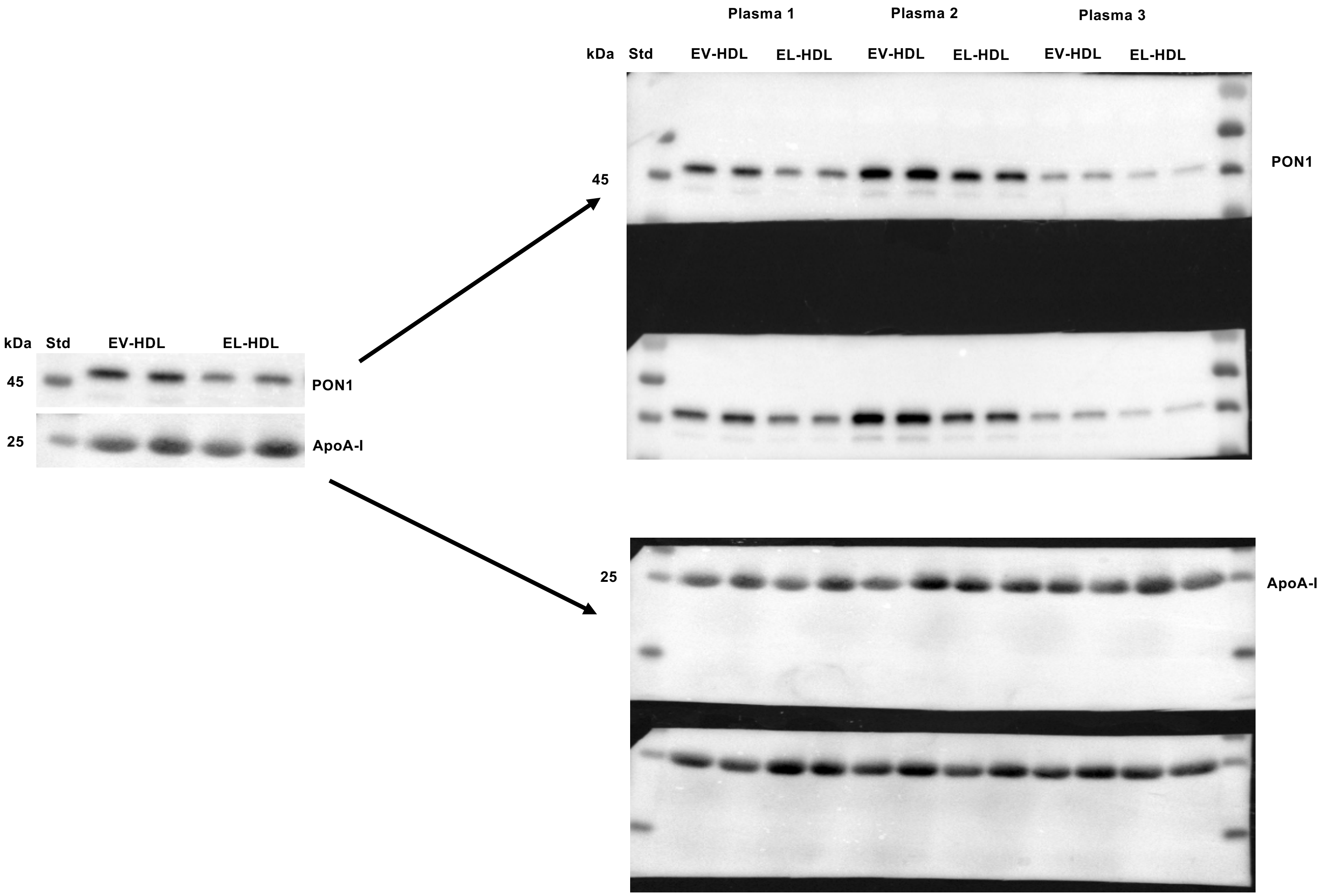
kDa
Std
EV-HDL
EL-HDL
45
PON1
25
ApoA-I
Plasma 1
Plasma 2
Plasma 3
kDa
Std
EV-HDL
EL-HDL
EV-HDL
EL-HDL
EV-HDL
EL-HDL
45
PON1
25
ApoA-I

Figure S5

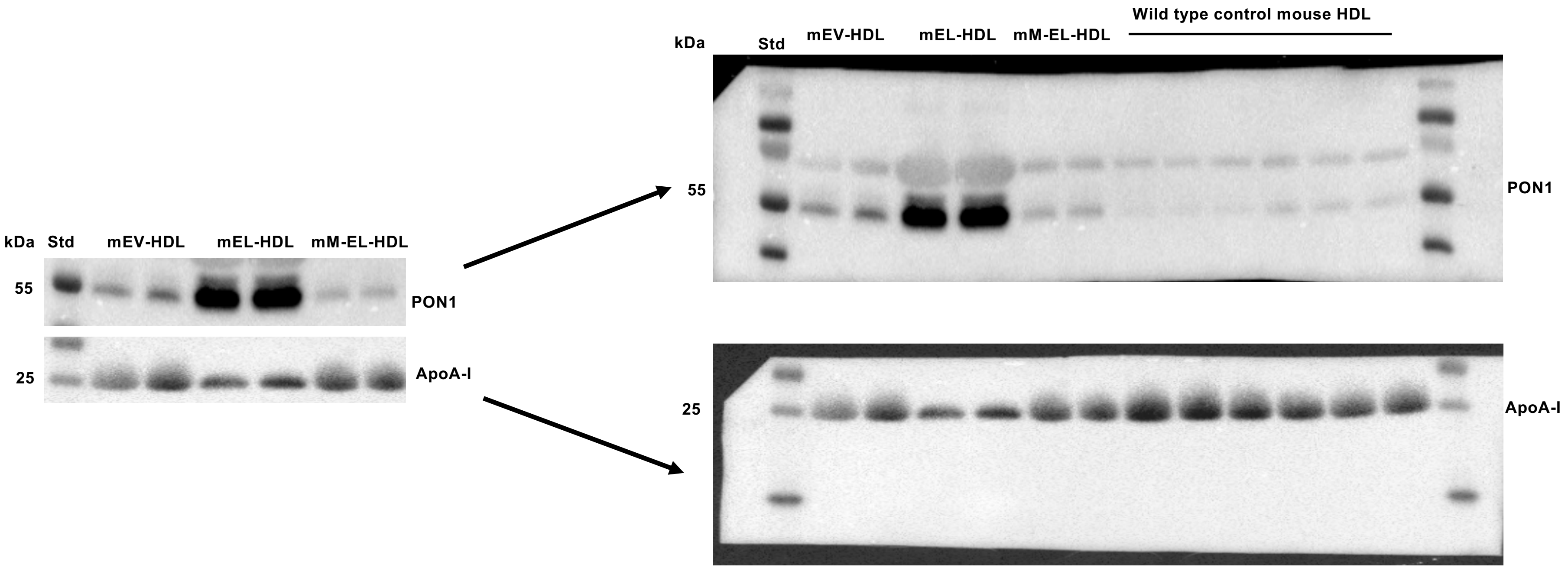
kDa
Std
mEV-HDL
mEL-HDL
mM-EL-HDL
55
PON1
25
ApoA-I
Wild type control mouse HDL

Figure S7
