# Supplementary material for: Endothelial Lipase Modulates Paraoxonase 1 Content and Arylesterase Activity of HDL
Source: Int J Mol Sci. 2021 Jan 13;22(2):719. doi: 10.3390/ijms22020719 (PMC7828365; doi:10.3390/ijms22020719)
Supplement: Supplementary file 1 [file ijms-22-00719-s001.zip › Suppl. Table S1.docx]

| Variable | Human samples  (n=42) |
| --- | --- |
| Age (years) | 40.9 (11.6) |
| Female (%) | 27 (64.3) |
| EL (pg/mL) mean (SD) 345.1 (96.9)  median (range) 340.9 (140.4-635.7) | |
| HDL PON1 mean (SD) 1694.2 (726.7)  (ng/mg HDL protein) median (range) 1606.0 (517.2 – 3317.2) | |
| HDL AE mean (SD) 38.0 (9.5)  (mmol/min/mg HDL median (range) 37.5 (20.7 – 56.9)  protein) | |
| Glucose (mmol/L) | 4.9 (0.5) |
| Bilirubin (µmol/L) | 9.3 (4.9) |
| Creatinine (µmol/L) | 69.5 (12.5) |
| Urea (mmol/L) | 4.5 (1.3) |
| Urate (µmol/L) | 285.4 (61.3) |
| GGT (U/L) | 19.6 (11.6) |
| AST (U/L) | 24.3 (7.6) |
| ALT (U/L) | 23.5 (8.7) |
| Protein (g/dL) | 7.2 (0.4) |
| Albumin (g/dL) | 4.7 (0.3) |
| Triacylglycerols (mmol/L) | 1.0 (0.5) |
| Total cholesterol (mmol/L) | 4.9 (0.8) |
| HDL cholesterol (mmol/L) | 1.8 (0.5) |
| LDL cholesterol (mmol/L) | 2.7 (0.6) |

**Table S1. Baseline characteristics of the blood donors**

Quantitative variables are presented as mean and standard deviation (SD); for EL, HDL PON1, and HDL AE median and range are additionally shown to highlight the cutoff value for ‘’high vs. low’’. For categorical variables the absolute (n) and relative frequencies (%) are reported. AE, arylesterase; EL, endothelial lipase; PON1, paraoxonase 1; GGT, gamma-glutamyl-transpeptidase; AST, aspartate aminotransferase, ALT, alanine aminotransferase; HDL, high-density lipoprotein; LDL, low-density lipoprotein.
