# Supplementary material for: Endothelial Lipase Modulates Paraoxonase 1 Content and Arylesterase Activity of HDL
Source: Int J Mol Sci. 2021 Jan 13;22(2):719. doi: 10.3390/ijms22020719 (PMC7828365; doi:10.3390/ijms22020719)
Supplement: Supplementary file 1 [file ijms-22-00719-s001.zip › Suppl. Table S10 .docx]

**Table S10.** Lipid species with levels significantly different in HDL isolated from high compared to low EL serum

| Lipid species  (pmol/µg HDL protein) | Low EL  (n=21) | High EL  (n=21) | Total  (n=42) | p-value |
| --- | --- | --- | --- | --- |
| CE 20:3 | 2.348 (0.517) | 2.674 (0.361) | 2.511 (0.470) | 0.024 |
| TAG 54:6 (18:3/36:3) | 0.128 (0.078) | 0.088 (0.044) | 0.108 (0.066) | 0.047 |
| LPC 22:3 | 0.012 (0.003) | 0.014 (0.003) | 0.013 (0.003) | 0.019 |
| LPC 22:4 | 0.015 (0.004) | 0.019 (0.005) | 0.017 (0.005) | 0.016 |
| LPE 22:3 | 0.116 (0.028) | 0.140 (0.042) | 0.128 (0.037) | 0.032 |
| LPE 22:4 | 0.106 (0.028) | 0.130 (0.040) | 0.118 (0.036) | 0.032 |
| PA 36:4 | 0.085 (0.025) | 0.068 (0.027) | 0.076 (0.027) | 0.046 |
| PG 34:2 | 0.108 (0.046) | 0.078 (0.035) | 0.093 (0.043) | 0.026 |
| PG 36:3 | 0.070 (0.038) | 0.050 (0.020) | 0.060 (0.032) | 0.034 |
| Cer d18:0/24:1 | 0.010 (0.004) | 0.012 (0.004) | 0.011 (0.004) | 0.044 |
| SM 36:1 | 4.027 (0.806) | 4.625 (1.027) | 4.326 (0.961) | 0.042 |

Data are presented as mean and standard deviation. The difference between high and low PON1 HDL samples was analyzed by unpaired t-test. For TAG, numbers in brackets refer to one elucidated fatty acid structure as determined by MS/MS fragmentation and the sum of the 2 other fatty acids.

EL, endothelial lipase; CE, cholesteryl ester, TAG, triacylglycerol; LPC, lysophosphatidylcholine; LPE, lysophosphatidylethanolamine, PA, phosphatidic acid; PG, phosphatidylglycerol; Cer, ceramide; SM, sphingomyelin; PON1, paraoxonase 1; HDL, high-density lipoprotein; MS, mass spectrometry.
