# Supplementary material for: Endothelial Lipase Modulates Paraoxonase 1 Content and Arylesterase Activity of HDL
Source: Int J Mol Sci. 2021 Jan 13;22(2):719. doi: 10.3390/ijms22020719 (PMC7828365; doi:10.3390/ijms22020719)
Supplement: Supplementary file 1 [file ijms-22-00719-s001.zip › Suppl. Table S11.docx]

**Table S11.** Significant associations between EL serum levels (high vs. low, determined by median) and HDL lipid species determined by univariable logistic regression analysis

| Lipid species  (pmol/µg HDL protein) | OR (95% CI) | p-value |
| --- | --- | --- |
| CE 20:3 | 2.27 (1.14-5.32) | 0.034 |
| LPC 22:3 | 2.30 (1.16-5.25) | 0.028 |
| LPC 22:4 | 2.40 (1.19-5.56) | 0.024 |
| LPE 22:3 | 2.12 (1.09-4.67) | 0.040 |
| LPE 22:4 | 2.12 (1.08-4.71) | 0.041 |
| PG 34:2 | 0.43 (0.17-0.88) | 0.040 |

ORs are presented per standard deviation increment.

EL, endothelial lipase; HDL, high-density lipoprotein; CE, cholesteryl ester; CI, confidence interval; LPC, lysophosphatidylcholine; LPE, lysophosphatidylethanolamine; PG, phosphatidylglycerol; Cer, ceramide; SM, sphingomyelin; PON1, paraoxonase 1; HDL, high-density lipoprotein; d, dihydro; OR, odds ratio.
