# Supplementary material for: Endothelial Lipase Modulates Paraoxonase 1 Content and Arylesterase Activity of HDL
Source: Int J Mol Sci. 2021 Jan 13;22(2):719. doi: 10.3390/ijms22020719 (PMC7828365; doi:10.3390/ijms22020719)
Supplement: Supplementary file 1 [file ijms-22-00719-s001.zip › Suppl. Table S2 .docx]

| Lipid species  (pmol/µg HDL protein) | Low PON1  (n=21) | High PON1  (n=21) | Total  (n=42) | p-value |
| --- | --- | --- | --- | --- |
| LPC 22:4 | 0.016 (0.004) | 0.019 (0.005) | 0.017 (0.005) | 0.018 |
| PA 32:1 | 0.007 (0.004) | 0.012 (0.009) | 0.009 (0.007) | 0.030 |
| PA 34:2 | 0.202 (0.116) | 0.288 (0.110) | 0.245 (0.120) | 0.019 |
| PA 36:1 | 0.014 (0.007) | 0.019 (0.009) | 0.016 (0.009) | 0.038 |
| PA 36:3 | 0.048 (0.028) | 0.085 (0.047) | 0.067 (0.043) | 0.005 |
| PG 38:3 | 0.026 (0.005) | 0.033 (0.011) | 0.029 (0.009) | 0.027 |
| PI 36:2 | 1.109 (0.374) | 1.440 (0.444) | 1.274 (0.439) | 0.013 |
| PI 38:3 | 0.532 (0.136) | 0.690 (0.276) | 0.611 (0.230) | 0.025 |
| Cer d18:2/22:0 | 0.012 (0.004) | 0.010 (0.003) | 0.011 (0.004) | 0.036 |
| Cer d18:2/23:0 | 0.011 (0.003) | 0.008 (0.002) | 0.010 (0.003) | 0.003 |
| Cer d18:2/23:1 | 0.00038 (0.00014) | 0.00025 (0.00009) | 0.00032 (0.00013) | 0.001 |
| Cer d18:2/24:0 | 0.038 (0.012) | 0.031 (0.009) | 0.034 (0.011) | 0.037 |
| Cer d18:2/24:1 | 0.020 (0.006) | 0.016 (0.003) | 0.018 (0.005) | 0.014 |
| Cer d18:2/24:2 | 0.00149 (0.00047) | 0.00119 (0.00033) | 0.00134 (0.00043) | 0.022 |
| Cer d18:2/26:0 | 0.00054 (0.00026) | 0.00040 (0.00012) | 0.00047 (0.00022) | 0.037 |
| Cer d18:2/26:1 | 0.00038 (0.00015) | 0.00028 (0.00009) | 0.00033 (0.00013) | 0.016 |

**Table S2.** Lipid species with levels significantly different in HDL with low compared to high PON1 (identified by logistic regression analysis)

Data are presented as mean and standard deviation. The difference between high and low PON1 HDL samples was analyzed by t-test.

LPC, lysophosphatidylcholine; PA, phosphatidic acid; PI, phosphatidylinositol; PG, phosphatidylglycerol; Cer, ceramide; PON1, paraoxonase 1; HDL, high-density lipoprotein.
