# Supplementary material for: Endothelial Lipase Modulates Paraoxonase 1 Content and Arylesterase Activity of HDL
Source: Int J Mol Sci. 2021 Jan 13;22(2):719. doi: 10.3390/ijms22020719 (PMC7828365; doi:10.3390/ijms22020719)
Supplement: Supplementary file 1 [file ijms-22-00719-s001.zip › Suppl. Table S3 .docx]

| Lipid species  (pmol/µg HDL protein) | low PON1/low EL | low PON1/high EL | p-value |
| --- | --- | --- | --- |
| DAG 18:2/18:2 | 0.089 (0.046) | 0.055 (0.026) | 0.048 |
| PA 36:4 | 0.083 (0.025) | 0.059 (0.018) | 0.027 |
| PG 34:2 | 0.112 (0.052) | 0.071 (0.028) | 0.027 |
| PG 36:3 | 0.069 (0.037) | 0.043 (0.019) | 0.047 |

**Table S3.** Levels of lipid species significantly enriched in low PON1/low EL compared to low PON1/high EL HDL

Data are presented as mean and standard deviation. The difference between groups was analyzed by unpaired t-test.

EL, endothelial lipase; DAG, diacylglycerol; PA, phosphatidic acid; PG, phosphatidylglycerol; PON1, paraoxonase 1; HDL, high-density lipoprotein.
