# Supplementary material for: Endothelial Lipase Modulates Paraoxonase 1 Content and Arylesterase Activity of HDL
Source: Int J Mol Sci. 2021 Jan 13;22(2):719. doi: 10.3390/ijms22020719 (PMC7828365; doi:10.3390/ijms22020719)
Supplement: Supplementary file 1 [file ijms-22-00719-s001.zip › Suppl. Table S4.docx]

| Lipid species  (pmol/µg HDL protein) | high PON1/low EL | high PON1/high EL | p-value |
| --- | --- | --- | --- |
| CE 18:1 | 143.5 (6.9) | 159.4 (18.5) | 0.011 |
| LPC 22:3 | 0.011 (0.004) | 0.015 (0.003) | 0.033 |
| LPE 22:3 | 0.100 (0.033) | 0.141 (0.040) | 0.024 |
| PI 38:2 | 0.053 (0.016) | 0.080 (0.037) | 0.031 |
| Cer d18:1/18:0 | 0.003 (0.001) | 0.004 (0.001) | 0.042 |
| Cer d18:2/18:0 | 0.001 (0.000) | 0.001 (0.000) | 0.042 |
| Cer d18:0/24:1 | 0.007 (0.003) | 0.012 (0.004) | 0.008 |
| SM 32:1 | 1.619 (0.312) | 1.998 (0.442) | 0.037 |
| SM 36:1 | 3.740 (0.485) | 4.608 (0.764) | 0.005 |

**Supplementary Table S4.** Levels of lipid species significantly enriched in high PON1/high EL compared to high PON1/low EL HDL

Data are presented as mean and standard deviation. The difference between groups was analyzed by unpaired t-test.

EL, endothelial lipase; CE, cholesteryl ester; Cer, ceramide; LPC, lysophosphatidylcholine; LPE, lysophosphatidylethanolamine; PI, phosphatidylinositol; SM, sphingomyelin; PON1, paraoxonase 1; HDL, high-density lipoprotein.
