# Supplementary material for: Endothelial Lipase Modulates Paraoxonase 1 Content and Arylesterase Activity of HDL
Source: Int J Mol Sci. 2021 Jan 13;22(2):719. doi: 10.3390/ijms22020719 (PMC7828365; doi:10.3390/ijms22020719)
Supplement: Supplementary file 1 [file ijms-22-00719-s001.zip › Suppl. Table S5.docx]

| HDL parameters  (mmol/L or g/L) | Low EL  (n=21) | High EL  (n=21) | Total  (n=42) | p-value |
| --- | --- | --- | --- | --- |
| HDL cholesterol | 1.880 (0.374) | 1.912 (0.378) | 1.896 (0.372) | 0.787 |
| HDL triacylglycerols | 0.123 (0.053) | 0.120 (0.053) | 0.121 (0.052) | 0.833 |
| HDL free cholesterol | 0.473 (0.123) | 0.488 (0.105) | 0.480 (0.114) | 0.669 |
| HDL phospholipids | 1.237 (0.253) | 1.252 (0.246) | 1.244 (0.247) | 0.851 |
| HDL apoA-I | 1.823 (0.295) | 1.820 (0.269) | 1.821 (0.279) | 0.977 |
| HDL apo A-II | 0.388 (0.063) | 0.378 (0.055) | 0.383 (0.059) | 0.585 |
| HDL1 triacylglycerols | 0.043 (0.022) | 0.044 (0.022) | 0.043 (0.022) | 0.925 |
| HDL2 triacylglycerols | 0.022 (0.012) | 0.021 (0.010) | 0.021 (0.011) | 0.767 |
| HDL3 triacylglycerols | 0.026 (0.013) | 0.025 (0.013) | 0.025 (0.013) | 0.726 |
| HDL4 triacylglycerols | 0.038 (0.013) | 0.036 (0.017) | 0.037 (0.015) | 0.728 |
| HDL1 cholesterol | 0.611 (0.253) | 0.675 (0.287) | 0.643 (0.270) | 0.448 |
| HDL2 cholesterol | 0.283 (0.071) | 0.288 (0.073) | 0.285 (0.071) | 0.829 |
| HDL3 cholesterol | 0.337 (0.063) | 0.335 (0.062) | 0.336 (0.062) | 0.902 |
| HDL4 cholesterol | 0.593 (0.102) | 0.575 (0.075) | 0.584 (0.089) | 0.524 |
| HDL1 free cholesterol | 0.185 (0.062) | 0.194 (0.066) | 0.190 (0.063) | 0.626 |
| HDL2 free cholesterol | 0.082 (0.019) | 0.080 (0.018) | 0.081 (0.018) | 0.736 |
| HDL3 free cholesterol | 0.094 (0.019) | 0.090 (0.015) | 0.092 (0.017) | 0.517 |
| HDL4 free cholesterol | 0.143 (0.023) | 0.134 (0.019) | 0.139 (0.021) | 0.212 |
| HDL1 phospholipids | 0.374 (0.149) | 0.400 (0.166) | 0.387 (0.156) | 0.590 |
| HDL2 phospholipids | 0.211 (0.060) | 0.212 (0.056) | 0.212 (0.057) | 0.957 |
| HDL3 phospholipids | 0.263 (0.056) | 0.258 (0.056) | 0.261 (0.055) | 0.810 |
| HDL4 phospholipids | 0.398 (0.058) | 0.387 (0.054) | 0.392 (0.055) | 0.558 |
| HDL1 apoA-I | 0.399 (0.164) | 0.419 (0.187) | 0.409 (0.174) | 0.709 |
| HDL2 apo A-I | 0.232 (0.059) | 0.230 (0.057) | 0.231 (0.057) | 0.887 |
| HDL3 apo A-I | 0.328 (0.066) | 0.322 (0.065) | 0.325 (0.064) | 0.747 |
| HDL4 apo A-I | 0.841 (0.121) | 0.824 (0.097) | 0.833 (0.108) | 0.601 |
| HDL1 apo A-II | 0.039 (0.018) | 0.040 (0.018) | 0.040 (0.018) | 0.895 |
| HDL2 apo A-II | 0.046 (0.015) | 0.045 (0.012) | 0.046 (0.013) | 0.879 |
| HDL3 apo A-II | 0.082 (0.021) | 0.080 (0.019) | 0.081 (0.020) | 0.746 |
| HDL4 apo A-II | 0.216 (0.034) | 0.207 (0.034) | 0.212 (0.034) | 0.383 |

**Supplementary Table S5**. Serum HDL parameters measured by NMR spectroscopy compared between high and low EL

Data are presented as mean and standard deviation. The difference between low and high EL samples was analyzed by unpaired t-test. HDL apo A-I and apo A-II parameters are given in g/dL and the other in mmol/L.

Densities of the HDL subclasses in kg/L: HDL1 (1.063-1.100), HDL2 (1.100-1.112), HDL3 (1.112-1.125), and HDL4 (1.125-1.210).

EL, endothelial lipase, NMR, nuclear magnetic resonance; HDL, high-density lipoprotein; apo, apolipoprotein.
