# Supplementary material for: Endothelial Lipase Modulates Paraoxonase 1 Content and Arylesterase Activity of HDL
Source: Int J Mol Sci. 2021 Jan 13;22(2):719. doi: 10.3390/ijms22020719 (PMC7828365; doi:10.3390/ijms22020719)
Supplement: Supplementary file 1 [file ijms-22-00719-s001.zip › Suppl. Table S6.docx]

**Table S6**. Correlation analysis of EL with serum HDL parameters measured by NMR spectroscopy

| HDL parameters | r | p-value |
| --- | --- | --- |
| HDL cholesterol | -0.033 | 0.837 |
| HDL triacylglycerols | 0.107 | 0.500 |
| HDL free cholesterol | -0.111 | 0.483 |
| HDL phospholipids | 0.047 | 0.766 |
| HDL apoA-I | 0.056 | 0.724 |
| HDL apo A-II | 0.114 | 0.474 |
| HDL1 triacylglycerols | 0.169 | 0.286 |
| HDL2 triacylglycerols | 0.098 | 0.536 |
| HDL3 triacylglycerols | 0.094 | 0.552 |
| HDL4 triacylglycerols | 0.064 | 0.686 |
| HDL1 cholesterol | -0.084 | 0.599 |
| HDL2 cholesterol | 0.003 | 0.987 |
| HDL3 cholesterol | -0.047 | 0.768 |
| HDL4 cholesterol | 0.004 | 0.978 |
| HDL1 free cholesterol | -0.065 | 0.684 |
| HDL2 free cholesterol | 0.025 | 0.874 |
| HDL3 free cholesterol | -0.066 | 0.680 |
| HDL4 free cholesterol | -0.049 | 0.756 |
| HDL1 phospholipids | -0.042 | 0.793 |
| HDL2 phospholipids | 0.039 | 0.807 |
| HDL3 phospholipids | -0.031 | 0.847 |
| HDL4 phospholipids | 0.021 | 0.894 |
| HDL1 apoA-I | -0.021 | 0.893 |
| HDL2 apo A-I | 0.118 | 0.456 |
| HDL3 apo A-I | -0.044 | 0.784 |
| HDL4 apo A-I | 0.109 | 0.492 |
| HDL1 apo A-II | 0.098 | 0.536 |
| HDL2 apo A-II | 0.146 | 0.355 |
| HDL3 apo A-II | -0.059 | 0.711 |
| HDL4 apo A-II | 0.044 | 0.784 |

Data presented are the Spearman correlation coefficient r, and the corresponding p-value.

The analysis comprised 42 serum samples.

EL, endothelial lipase, NMR, nuclear magnetic resonance; HDL, high-density lipoprotein; apo, apolipoprotein.
