# Supplementary material for: Endothelial Lipase Modulates Paraoxonase 1 Content and Arylesterase Activity of HDL
Source: Int J Mol Sci. 2021 Jan 13;22(2):719. doi: 10.3390/ijms22020719 (PMC7828365; doi:10.3390/ijms22020719)
Supplement: Supplementary file 1 [file ijms-22-00719-s001.zip › Suppl. Table S7.docx]

| Lipid species  (pmol/µg HDL protein) | OR (95% CI) | p-value |
| --- | --- | --- |
| DAG 16:0/18:0 | 0.31 (0.09-0.80) | 0.039 |
| DAG 18:0/18:0 | 0.36 (0.12-0.83) | 0.036 |
| PC 30:1 | 0.47 (0.20-0.93) | 0.048 |
| PC 36:1 | 0.49 (0.23-0.95) | 0.048 |
| PG 34:2 | 0.44 (0.17-0.90) | 0.043 |
| Cer d18:2/23:0 | 0.44 (0.20-0.86) | 0.024 |
| Cer d18:2/23:1 | 0.39 (0.17-0.78) | 0.015 |
| Cer d18:2/24:0 | 0.44 (0.19-0.88) | 0.034 |
| Cer d18:2/24:1 | 0.32 (0.12-0.70) | 0.011 |
| Cer d18:2/24:2 | 0.49 (0.23-0.94) | 0.040 |
| Cer d18:2/26:1 | 0.40 (0.16-0.83) | 0.027 |
| SM 30:1 | 0.39 (0.15-0.81) | 0.025 |
| SM 32:1 | 0.46 (0.21-0.90) | 0.034 |

**Table S7.** Significant associations between HDL AE activity and HDL lipid species determined by univariable logistic regression analysis

ORs are presented per standard deviation increment.

AE, arylesterase; DAG, diacylglycerol; PC, phosphatidylcholine; PG, phosphatidylglycerol; Cer, ceramide; SM, sphingomyelin; PON1, paraoxonase 1; HDL, high-density lipoprotein; d, dihydro; OR, odds ratio; CI, confidence interval.
