# Supplementary material for: Endothelial Lipase Modulates Paraoxonase 1 Content and Arylesterase Activity of HDL
Source: Int J Mol Sci. 2021 Jan 13;22(2):719. doi: 10.3390/ijms22020719 (PMC7828365; doi:10.3390/ijms22020719)
Supplement: Supplementary file 1 [file ijms-22-00719-s001.zip › Suppl. Table S8.docx]

| Lipid species  (pmol/µg HDL protein) | Low AE  (n=21) | High AE  (n=21) | Total  (n=42) | p-value |
| --- | --- | --- | --- | --- |
| DAG 16:0/18:0 | 0.065 (0.044) | 0.039 (0.021) | 0.052 (0.037) | 0.023 |
| DAG 18:0/18:0 | 0.073 (0.041) | 0.049 (0.023) | 0.061 (0.035) | 0.025 |
| PC 30:1 | 0.158 (0.098) | 0.105 (0.053) | 0.132 (0.082) | 0.037 |
| PC 36:1 | 14.0 (3.2) | 12.2 (1.9) | 13.1 (2.8) | 0.041 |
| PG 34:2 | 0.108 (0.050) | 0.078 (0.029) | 0.093 (0.043) | 0.031 |
| Cer d18:2/23:0 | 0.011 (0.003) | 0.009 (0.003) | 0.010 (0.003) | 0.017 |
| Cer d18:2/23:1 | 0.00037 (0.00013) | 0.00026 (0.00012) | 0.00032 (0.00013) | 0.008 |
| Cer d18:2/24:0 | 0.038 (0.012) | 0.030 (0.009) | 0.034 (0.011) | 0.025 |
| Cer d18:2/24:1 | 0.020 (0.005) | 0.016 (0.004) | 0.018 (0.005) | 0.004 |
| Cer d18:2/24:2 | 0.00148 (0.00040) | 0.00120 (0.00041) | 0.00134 (0.00043) | 0.034 |
| Cer d18:2/26:1 | 0.00038 (0.00014) | 0.00028 (0.00011) | 0.00033 (0.00013) | 0.016 |
| SM 30:1 | 0.106 (0.036) | 0.081 (0.024) | 0.094 (0.033) | 0.013 |
| SM 32:1 | 2.081 (0.493) | 1.756 (0.413) | 1.919 (0.478) | 0.026 |

**Table S8.** Lipid species with levels significantly different in HDL with high compared to low AE activity

Data are presented as mean and standard deviation. The difference between low and high AE samples was analyzed by unpaired t-test.

AE, arylesterase; DAG, diacylglycerol; PC, phosphatidylcholine; PG, phosphatidylglycerol; Cer, ceramide; SM, sphingomyelin; PON1, paraoxonase 1; HDL, high-density lipoprotein; d, dihydro;
