# Supplementary material for: Endothelial Lipase Modulates Paraoxonase 1 Content and Arylesterase Activity of HDL
Source: Int J Mol Sci. 2021 Jan 13;22(2):719. doi: 10.3390/ijms22020719 (PMC7828365; doi:10.3390/ijms22020719)
Supplement: Supplementary file 1 [file ijms-22-00719-s001.zip › Suppl. table S9.docx]

**Table S9**. Significant correlations of AE activity with HDL lipid species

| Lipid species | r | p-value |
| --- | --- | --- |
| CE 18:1 | -0.309 | 0.047 |
| TAG 48:0 (16:0/32:0) | -0.314 | 0.043 |
| TAG 48:3 (14:0/34:3) | -0.309 | 0.047 |
| TAG 50:1 (14:0/36:1) | -0.328 | 0.035 |
| TAG 52:1 (18:1/34:0) | -0.345 | 0.026 |
| TAG 54:2 (18:0/36:2) | -0.317 | 0.042 |
| PA 34:1 | -0.400 | 0.009 |
| PC 30:2 | -0.388 | 0.012 |
| PC 34:0 | -0.379 | 0.014 |
| PC 34:1 | -0.384 | 0.013 |
| PC 36:1 | -0.395 | 0.010 |
| PC 36:4 | -0.315 | 0.043 |
| PE 36:1 | -0.323 | 0.037 |
| PE 38:5 | -0.311 | 0.045 |
| PG 34:2 | -0.346 | 0.027 |
| PI 38:2 | -0.355 | 0.021 |
| PI 40:4 | -0.376 | 0.015 |
| Cer d18:1/14:0 | -0.316 | 0.042 |
| Cer d18:2/16:0 | -0.333 | 0.032 |
| Cer d18_2_18_0 | -0.316 | 0.042 |
| Cer d18:2/22:0 | -0.326 | 0.036 |
| Cer d18:2/23:0 | -0.317 | 0.041 |
| Cer d18:2/23:1 | -0.364 | 0.018 |
| Cer d18:2/24:0 | -0.315 | 0.042 |
| Cer d18:2/24:1 | -0.354 | 0.022 |
| SM 30:1 | -0.354 | 0.022 |
| SM 32:1 | -0.420 | 0.006 |
| SM 34:1 | -0.380 | 0.013 |
| SM 34:2 | -0.416 | 0.007 |
| SM 36:1 | -0.335 | 0.031 |
| SM 38:1 | -0.325 | 0.036 |
| SM 40:2 | -0.431 | 0.005 |

Data presented are the Spearman correlation coefficient r and the corresponding p-value.

The analysis comprised 42 samples. For TAG, numbers in brackets refer to one elucidated fatty acid structure as determined by MS/MS fragmentation and the sum of the 2 other fatty acids.

CE, cholesteryl ester; TAG, triacylglycerol; PA, phosphatidic acid; PC, phosphatidylcholine; PE, phosphatidylethanolamine; PG, phosphatidylglycerol; PI, phosphatidylinositol; Cer, ceramide; SM, sphingomyelin; MS, mass spectrometry.
