# Supplementary material for: Endothelial Lipase Modulates Paraoxonase 1 Content and Arylesterase Activity of HDL
Source: Int J Mol Sci. 2021 Jan 13;22(2):719. doi: 10.3390/ijms22020719 (PMC7828365; doi:10.3390/ijms22020719)
Supplement: Supplementary file 1 [file ijms-22-00719-s001.zip › Suppl.Figs.pdf]

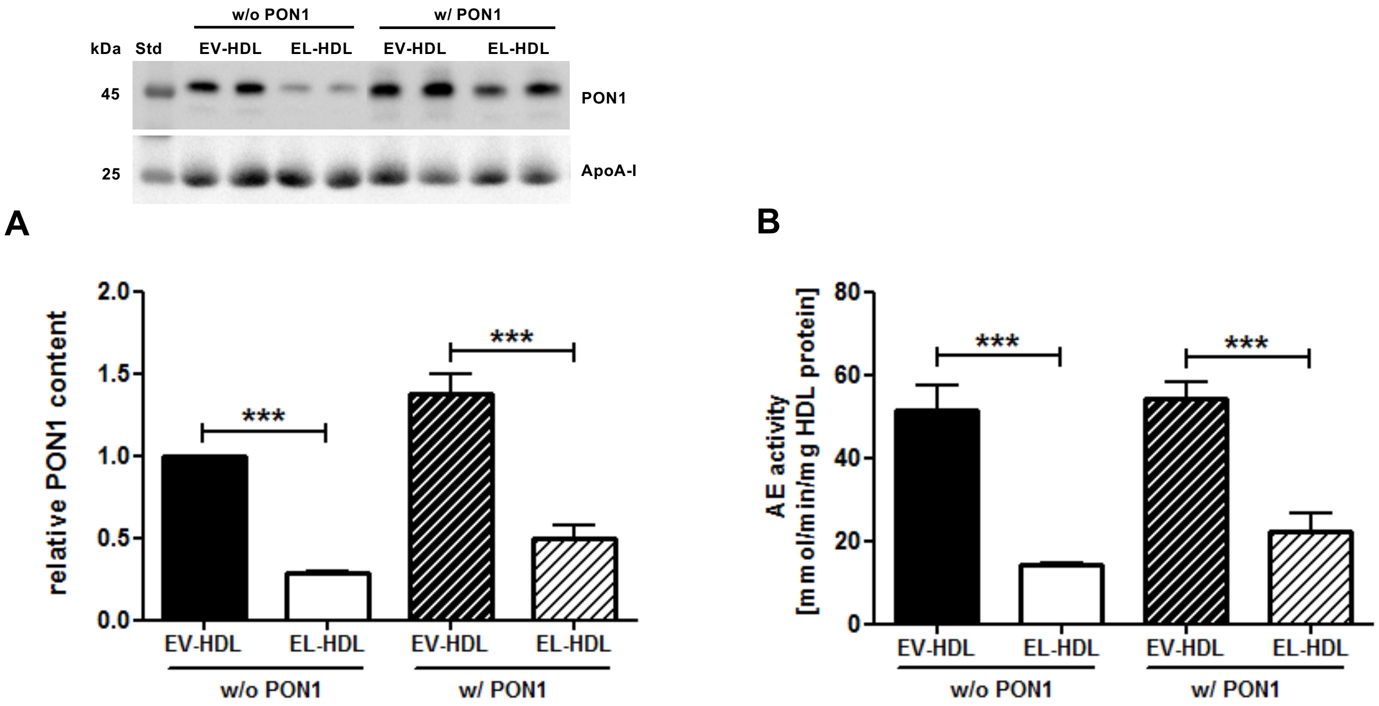

Figure S1. EL decreases HDL PON1 content and AE activity in the presence of bovine serum albumin (BSA)

EV-HDL and EL-HDL were generated by incubation of human HDL with EV or EL overexpressing cells in the absence (w/o) or presence (w/) of PON1 overexpression and in the presence of 4% BSA under cell culture conditions for 16 h. Modified HDL was purified by ultracentrifugation. (A) 10 µg HDL protein were separated by 12% SDS-PAGE followed by Western blotting analyses of PON1 and apoA-I. (B) 2 µg HDL protein were used for measurement of PON1 AE activity. Densitometric values of PON1 were normalized to the corresponding apoA-I signals. (B) PON1 AE activity was measured in 2 µg HDL protein. Results are presented as means + SEM of 3 independent modifications of human HDL each loaded onto a gel (Western blotting) or measured (AE activity) in duplicate. The difference between EL-HDL and EV-HDL was analyzed by two-tailed unpaired t-test in samples w/ and w/o PON1 separately. ***$P < 0.001$.

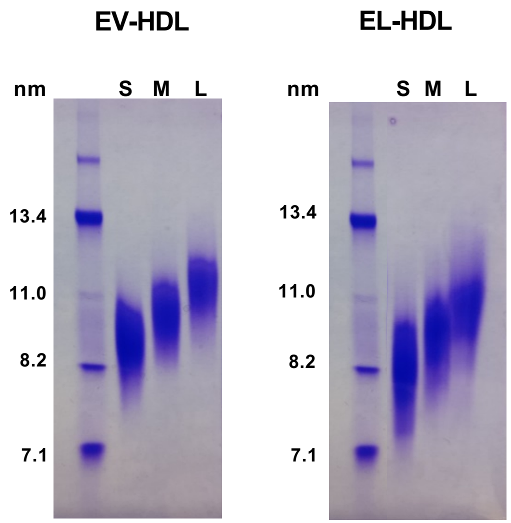

Figure S2. FPLC-derived EV-HDL- and EL-HDL-fractions

EV-HDL and EL-HDL were fractionated by FPLC to obtain small (S), medium (M), and large (L) EV-HDL and EL-HDL particles. 10 µg HDL protein were separated by 4-20% non-denaturing polyacrylamide gels followed by Coomassie Blue staining.

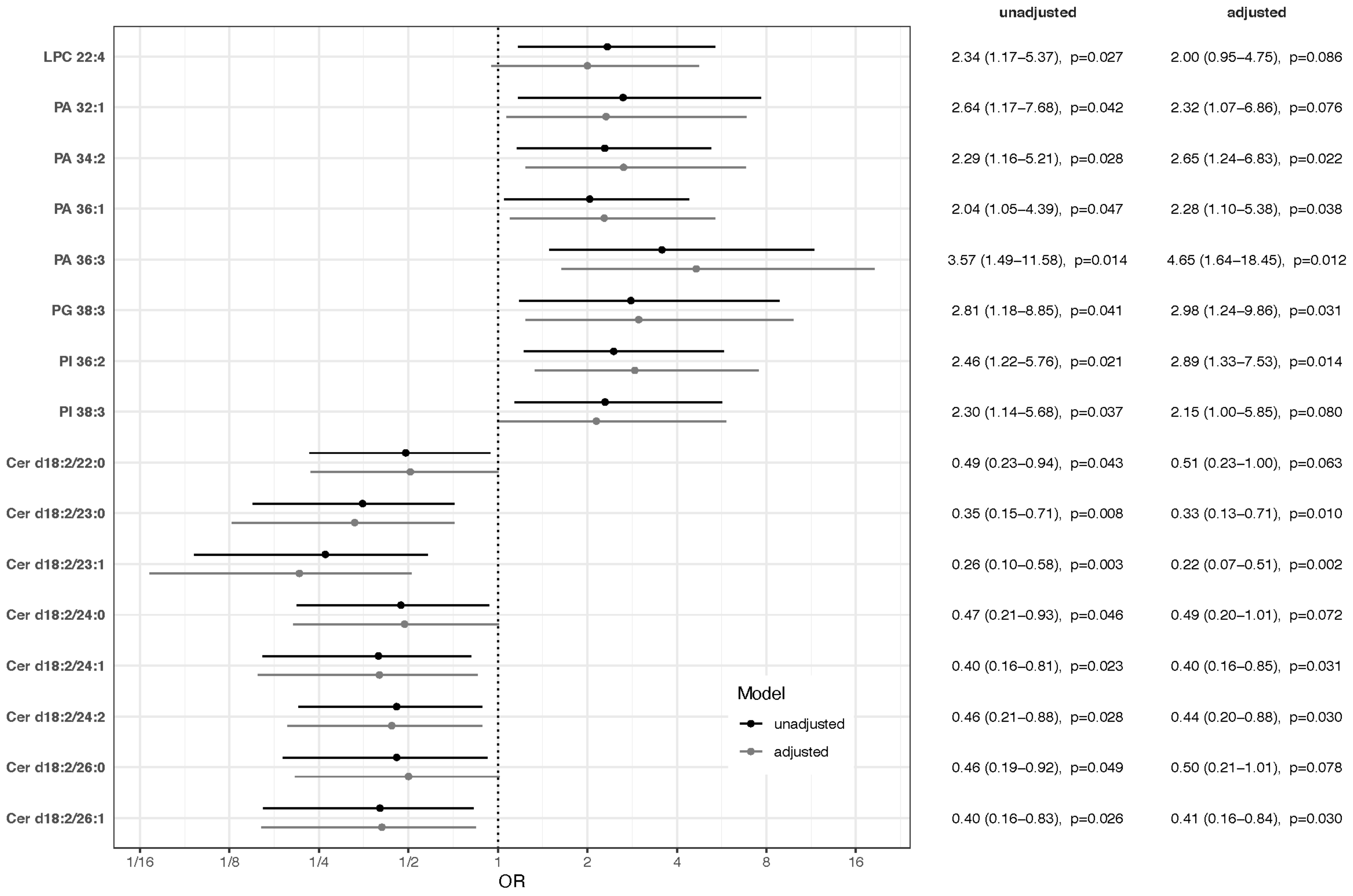

Figure S3. Association between HDL PON1 content and lipid composition is minimally affected by EL

OR and CI correspond to 1 SD increments and are presented on the log-2-scale; SDs for the lipid species are shown in Suppl. Table 2. Data of 42 subjects were available for univariable as well as for the analysis adjusted for EL serum levels.

CI, confidence interval; LPC, lysophosphatidylcholine; PA, phosphatidic acid; PI, phosphatidylinositol; PG, phosphatidylglycerol; Cer, ceramide; OR, odds ratio; SD, standard deviation.

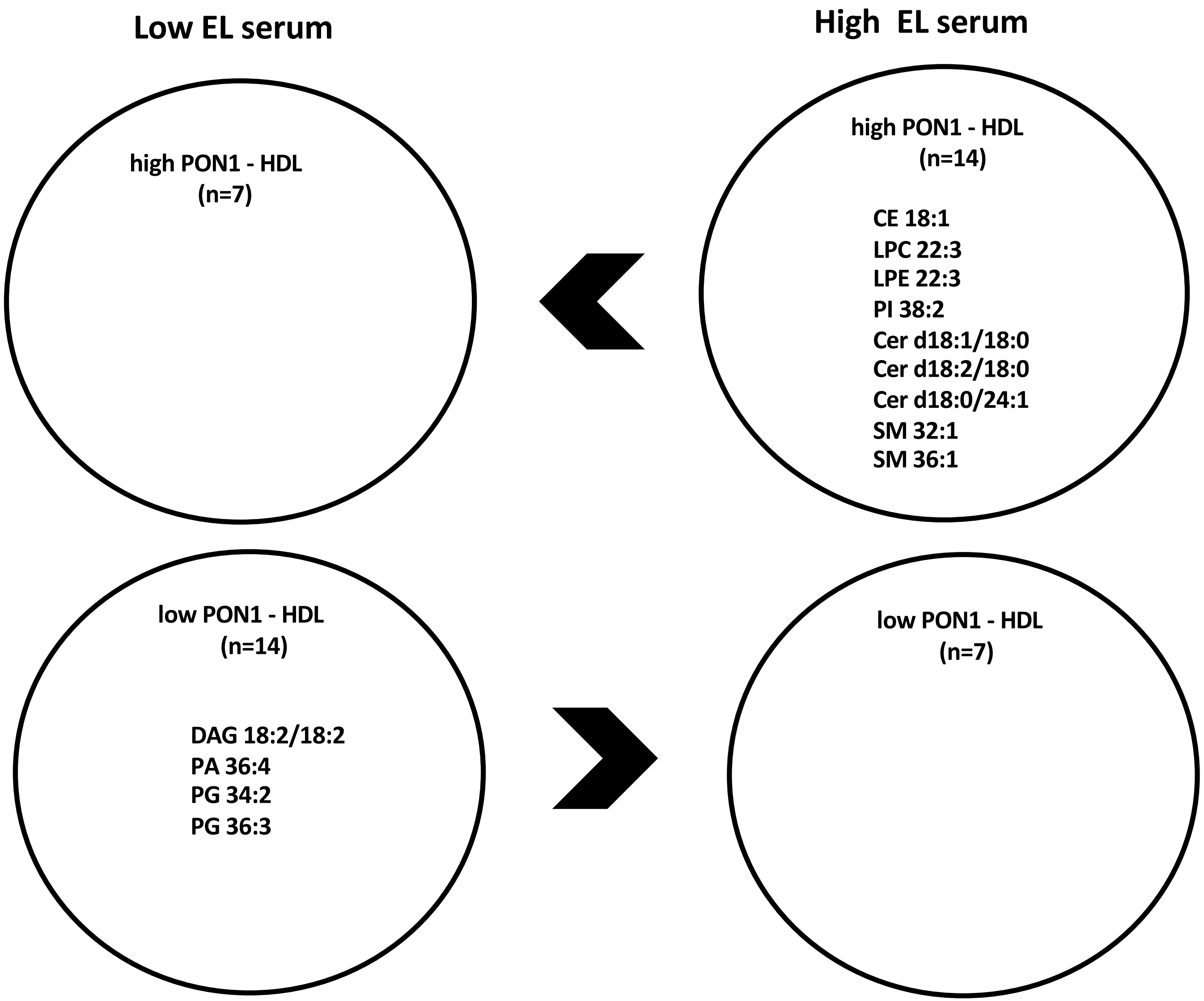

Figure S4. HDL PON1 content is not determined by EL-related lipidomic signature of HDL

HDL lipid composition was determined by mass spectrometry. Presented are 4 different subsets of HDL (circles) defined by PON1 content and EL serum concentrations: high PON1/high EL, high PON1/low EL, low PON1/high EL, and low PON1/low EL. The levels of lipid species indicated in a particular HDL are significantly higher compared to the HDL pointed with an arrow. Related quantitative data are shown in Tables S3 and S4.

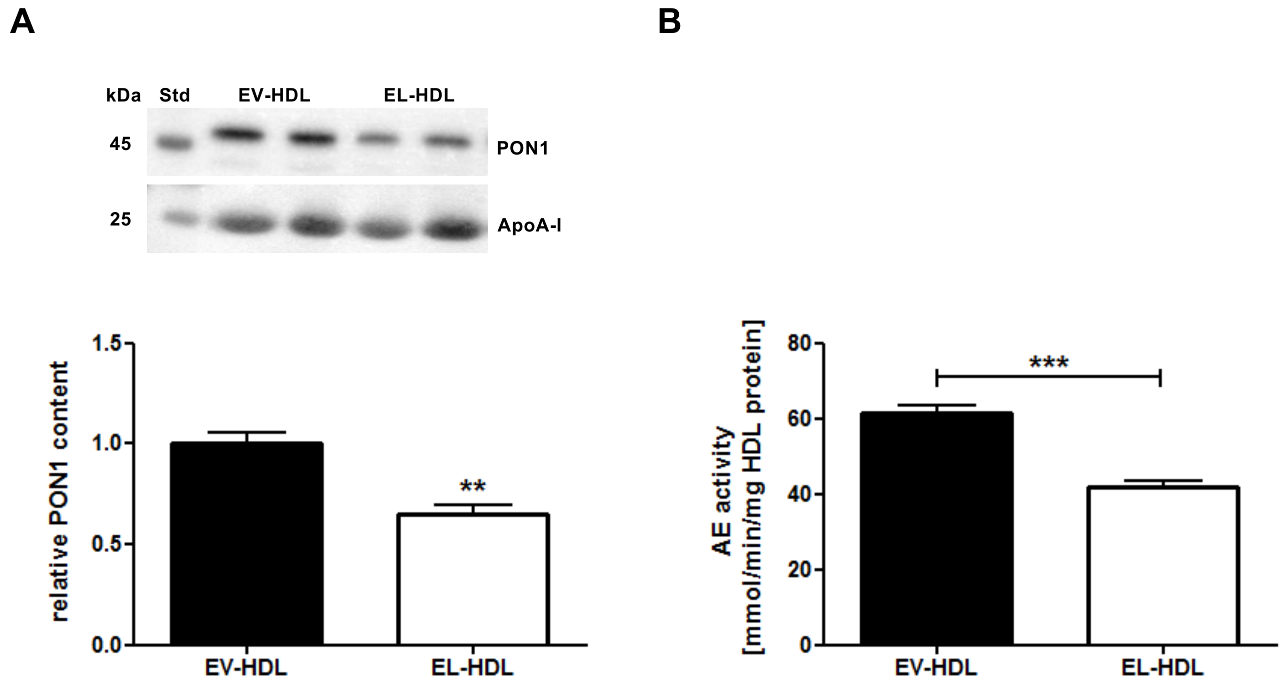

Figure S5 EL-modification of human plasma *in vitro* decreases HDL PON1 content and AE activity

EV-HDL and EL-HDL were generated by incubation of 50% human plasma with EV- or EL- overexpressing cells under cell culture conditions for 16 h. Modified HDL was purified by ultracentrifugation. (A) 10 µg HDL protein were separated by 12% SDS-PAGE followed by Western blotting analyses of PON1 and apoA-I. Densitometric values of PON1 were normalized to the corresponding apoA-I signals. (B) 2 µg HDL protein were used for measurement of PON1 AE activity. Results obtained by densitometry and measurements of AE activity are presented as means + SEM of 3 independent modifications of human HDL each loaded onto gel (Western blotting) or measured (AE activity) in duplicate. The difference between EL-HDL and EV-HDL was analyzed by two-tailed unpaired t-test. ** $P < 0.01$, *** $P < 0.001$.

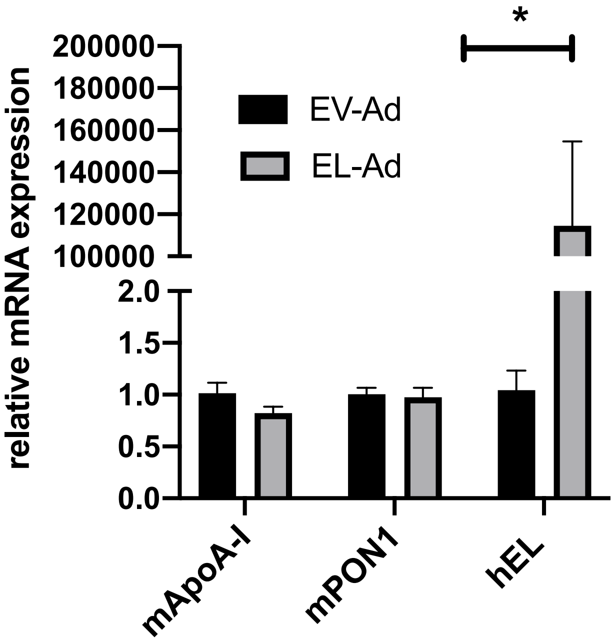

Figure S6. Mouse liver apoA-I and PON1 mRNA levels are unaltered upon overexpression of human EL in mice

Total RNA was isolated from the liver of 4 EV-Adenovirus (Ad)- and 5 EL-Ad-transduced mice followed by determination of mouse (m) apoA-I and PON1 as well as human (h) EL mRNA expression. mRNA expression was analyzed in duplicates by real-time PCR and normalized to cyclophilin A expression as a reference gene. Expression profiles were determined by the $2^{-\Delta\Delta CT}$ method. * $P < 0.05$.

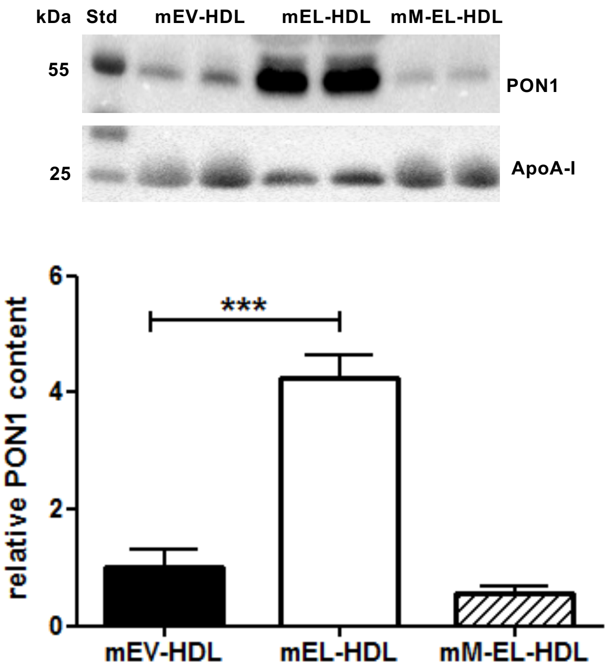

Figure S7. HDL PON1 content is unaltered upon overexpression of enzymatically inactive EL (M-EL) in mice

HDL was isolated by ultracentrifugation from serum of EV-, EL-, and M-EL-overexpressing mice 48 h after adenovirus (Ad) injection. 10 µg HDL protein were separated by 12% SDS-PAGE followed by Western blotting analyses of PON1 and apoA-I. Densitometry results are mean + SEM of 3 independent *in vivo* modifications, with 10 EL-Ad, 4 M-EL-Ad, and 4 EV-Ad-injected mice per each modification. Samples from each modification were loaded onto gels in duplicate. The difference between EL-HDL and EV-HDL as well as between M-EL-HDL and EV-HDL was analyzed by two-tailed unpaired t-test. ***$P < 0.001$.
